# Early postoperative outcomes in a retrospective propensity score-matched comparison of robotic extended totally extraperitoneal (r-eTEP) and extended totally extraperitoneal (eTEP) repair for ventral hernia

**DOI:** 10.1007/s10029-025-03293-z

**Published:** 2025-03-12

**Authors:** Asem Al-Salemi, Nader El-Sourani, Maximilian Bockhorn, Fadl Alfarawan

**Affiliations:** 1 Department for General and Visceral Surgery, University Hospital Oldenburg, Klinikum Oldenburg AöR, Rahel-Straus-Straße 10, 26133 Oldenburg, Germany; 2 Carl von Ossietzky Universität Oldenburg Fakultät VI - Medizin und Gesundheitswissenschaften, Ammerländer Heerstraße 114-118, 26129 Oldenburg, Germany; 3 Department for General-, Visceral– and Transplantation Surgery, University Hospital Münster, Albert-Schweitzer-Campus 1, 48149 Münster, Germany

**Keywords:** eTEP, r-eTEP, Robotic, Ventral hernias, extraperitoneal

## Abstract

**Background:**

The extended totally extraperitoneal technique (eTEP) is a novel approach for ventral hernia repair. This technique has been recently advanced using robotics (r-eTEP). The aim of this study is to perform a comprehensive analysis of the initial results of r-eTEP and to evaluate the safety and efficacy of this technique compared to the eTEP technique.

**Methods:**

This is a monocentric retrospective study of patients with ventral hernias who underwent surgery via eTEP or r-eTEP in our department between 2019 and 2023. Propensity score matching was applied to compare the groups. Preoperative patient and hernia characteristics, intraoperative findings, and postoperative outcomes were subsequently analysed.

**Results:**

Patient demographics were comparable between the groups. The r-eTEP group had a significantly greater proportion of M3 hernias (*p* = 0.006), M4 hernias (*p* = 0.020), incisional hernias (*p* = 0.002), and hernias with rectus diastasis (*p* < 0.001). The r-eTEP group had a significantly larger hernia defect (*p* = 0.003) and larger mesh size (*p* = 0.015). The r-eTEP group had a shorter hospital stay (*p* < 0.001) and shorter operative time, though not statistically significant (*p* = 0.211). Intraoperative and postoperative complications, as well as postoperative pain, were comparable between the groups.

**Conclusion:**

The findings of the present study show that the r-eTEP technique may offer potential benefits as the overall hospital stay was shorter while intraoperative and postoperative complications were comparable for both techniques.

## Introduction

Ventral hernias, defined as a protrusion of abdominal content through a defect in the fascia of the abdominal wall [1] encompassing congenital and acquired defects, are a common condition associated with significant patient morbidity, including paint, incarceration, and recurrence [2–6].

Ventral hernia repair (VHR) is one of the most frequently performed procedures in the United States, with over 350,000 operations annually [7]. Various techniques for VHR exist, ranging from open procedures with or without mesh placement [8] to minimally invasive approaches such as laparoscopic and robotic-assisted techniques.

Minimally invasive methods have demonstrated lower complication rates, shorter hospital stays, lower treatment costs and a reduced risk of recurrence compared to open repair [9–12]. Among these, robotic surgery offers additional benefits including enhanced precision and ergonomics [13, 14].

The extended totally extraperitoneal repair (eTEP), initially introduced for inguinal hernia repair, has been adapted for ventral and incisional hernias, yielding promising results such as low complication rates, reduced postoperative pain and shorter hospital stay [15–18]. Compared to other minimally invasive methods (i.e. intraperitoneal onlay mesh) eTEP is associated with lower odds of hernia recurrence and a lower risk of surgical site infection (SSI) as demonstrated in multiple meta-analysis and systematic reviews [19].

Robotic eTEP (r-eTEP) combines the advantages of robotic platforms with the benefits of eTEP, allowing for precise, minimally invasive placement of the retromuscular mesh. Furthermore it can be combined with a transversus abdominis release (TAR) [20, 21]. TAR is one of the only techniques allowing primary closure of large defects while simultaneously maintaining retromuscular mesh placement. This is of utmost importance as primary fascial closure is associated with a lower hernia recurrence [22]. Compared with laparoscopic ventral hernia repair (lapVHR), robot-assisted ventral hernia repair (rVHR) shows improved results with a shorter hospital length of stay, lower reoperation rate, fewer conversions to laparotomy and comparable complication and recurrence rates [23–27], although concerns remain regarding longer operative times and higher costs [25, 28].

The comparison between eTEP and r-eTEP is important to understand the advantages and disadvantages of each technique and to make evidence-based recommendations for the selection of the optimal surgical method. In addition, due to the newly introduced technique and the limited number of studies on this topic, further studies are needed. To date, only a limited number of studies have analysed r-eTEP, and to our knowledge, only one study has compared eTEP and r-eTEP [20, 21, 29–31]. In their comparison of both techniques, Lu et al. reported that r-eTEP is used more frequently in patients with more complex abdominal wall defects and has similar perioperative outcomes, such as length of stay and reoperation rates, but with significantly longer operating times and higher hospital costs [32]. However, propensity score matching (PSM) was not conducted, limiting the ability to balance group characteristics for accurate outcome comparisons.

The purpose of this study is to present and provide a comprehensive analysis of early postoperative results of r-eTEP and compare operative outcomes between r-eTEP and laparoscopic eTEP through a propensity score matched analysis.

## Methods

### Study population and design

This monocentric retrospective study analysed data from a prospective database of patients with ventral hernias who underwent surgery between 2019 and 2023 at the Department for General and Visceral Surgery. Only patients treated using either eTEP or r-eTEP technique were included. Patients with inguinal hernias, patients, those who underwent surgery via the transabdominal preperitoneal hernia repair (TAPP) technique or other surgical techniques, and those lost to follow-up were excluded from the analysis.All procedures were performed by a single experienced surgeon to ensure consistency and minimize variability in surgical technique.

Inclusion criteria included primary ventral hernias located in the M1 to M5 areas, as well as patients with incisional hernias in the same regions, including those with defect sizes greater than 7 cm. In such cases, a CT scan was performed to plan the optimal surgical strategy. Additionally, Carbonell’s algorithm was applied to determine whether a transversus abdominis release (TAR) was necessary [33].

Exclusion criteria encompassed primary ventral hernias located in the L1 to L4 areas and incisional hernias in the same regions. Further exclusions included a history of sublay mesh implantation, open abdomen (abdominis apertum), and gastrointestinal fistulas, re-do or reccurence operation.

For laparoscopic eTEP, additional exclusion criteria were applied, including defect sizes greater than 7 cm and a history of previous transverse laparotomy.

All patients indicated for eTEP were included in the study. In the initial phase (until June 2023), the robotic platform was unavailable, so all procedures were performed laparoscopically. From June 2023 onwards, when the robotic platform became available, all eTEP procedures were performed with robotic assistance. The choice of surgical technique was solely determined by the availability of the robotic platform, not by hernia-specific factors.

### Data collection

The clinical dataset included demographic and clinical variables such as age, sex, body mass index as kg/m^2^ (BMI), American Society of Anesthesiologists (ASA) classification, preoperative risk factors, and comorbidities, Hernia characteristics were documented, including type (classified using the European Hernia Society (EHS) classification) [2], defect size (cm^2^) and implanted mesh size (cm^2^).

### Perioperative and postoperative data

Intraoperative data included operation time in minutes (skin-to-skin), size of hernia defect (cm^2^), mesh size (cm^2^), drain placement, conversion rate, and intraoperative complications. Postoperative complications were categorized according to the Clavien‒Dindo classification system [34] and included general complications, surgical site occurences (SSOs) and surgical site infections (SSIs) [35]. SSIs encompassed all superficial and deep infections, while SSOs included non-infectious, wound-specific complictions such as hematomas, seromas, wound dehiscence, delayed wound healing, or necrosis.

### Pain management and assessment

Postoperative pain was evaluated using the visual analogue scale (VAS), with scores recorded multiple times daily to account for the effects of pain medication. The average VAS score was used for data analysis. Pain management followed our hospital’s standardized protocol: oral metamizole (1000 mg) administered 4 to 6 times daily, with additional oral oxycodone/naloxone (5-10 mg) prescribed twice daily for VAS scores exceeding 4. For acute pain episodes, 10 mg of oral morphine was available on demand.

### Follow-up

A follow-up period of 30 days was established for the postoperative outcomes. A follow-up appointment was scheduled for all patients 30 days postoperatively, with earlier appointments arranged for patients who experienced complications.

### Surgical technique - laparoscopic eTEP

**Fig. 1:**
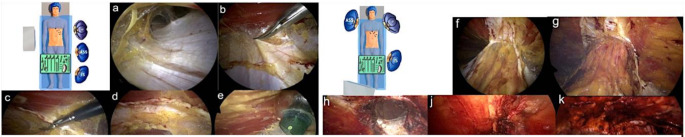
Laparoscopic eTEP (**a**) Blunt dissection of the retro muscular space; (**b**) Transection of the posterior fascial layer; (**c**) Preperitoneal dissection behind the linea alba; (**d**) Transection of the contralateral posterior fascial layer and opening of the retro rectal space; (**e**) Placement of the camera trocar in the contralateral retro muscular space; (**f**) Cranio caudal view of both retro-muscular and preperitoneal space; (**g**) Cranial depiction of the hernia; (**h**) Repositioning of the hernia sac and content; (**j**) Continuous closure of the defect; (**k**) Placement of the mesh

The following describes eTEP surgery of a lower midline hernia. The first 11-mm optical trocar is inserted into the retro muscular space. The trocar is placed along the rib margins, approximately on the medio clavicular line. After blunt dissection, the retro muscular space is insufflated with CO2 at 20 mmHg (Fig. 1a). Next, two additional 5-mm working trocars are inserted along the linea semilunaris. Dissection progresses toward the medial edge of the posterior layer of the rectus sheath, exposing the fusion between the anterior and posterior layers. A crossover is performed to create a connection between the preperitoneal space behind the linea alba and both retro muscular spaces (Fig. 1b-d). A fourth 11-mm trocar is placed on the opposite side of the midline (Fig. 1e), and the optic is switched to this trocar for improved visibility. Dissection continues from cranial to caudal. The hernia sac is identified, and its contents are reduced. Dissection is extended at least 5 cm below the hernia defect. (Fig. 1f-h). The size of the hernia is measured, and the defect is closed. The linea alba is reconstructed using a long-term absorbable, self-fixating Stratafix suture (0) (Fig. 1j). Any defects in the posterior fascial layer are closed with 2 − 0 Stratafix sutures. After measuring the prepared space, a biocompatible mesh (Soft-Mesh) is inserted. If necessary, a drain may be placed. Finally, while ensuring correct positioning of the mesh the gas is released from the retro muscular space (Fig. 1k).

### Surgical technique - robotic eTEP

**Fig. 2:**
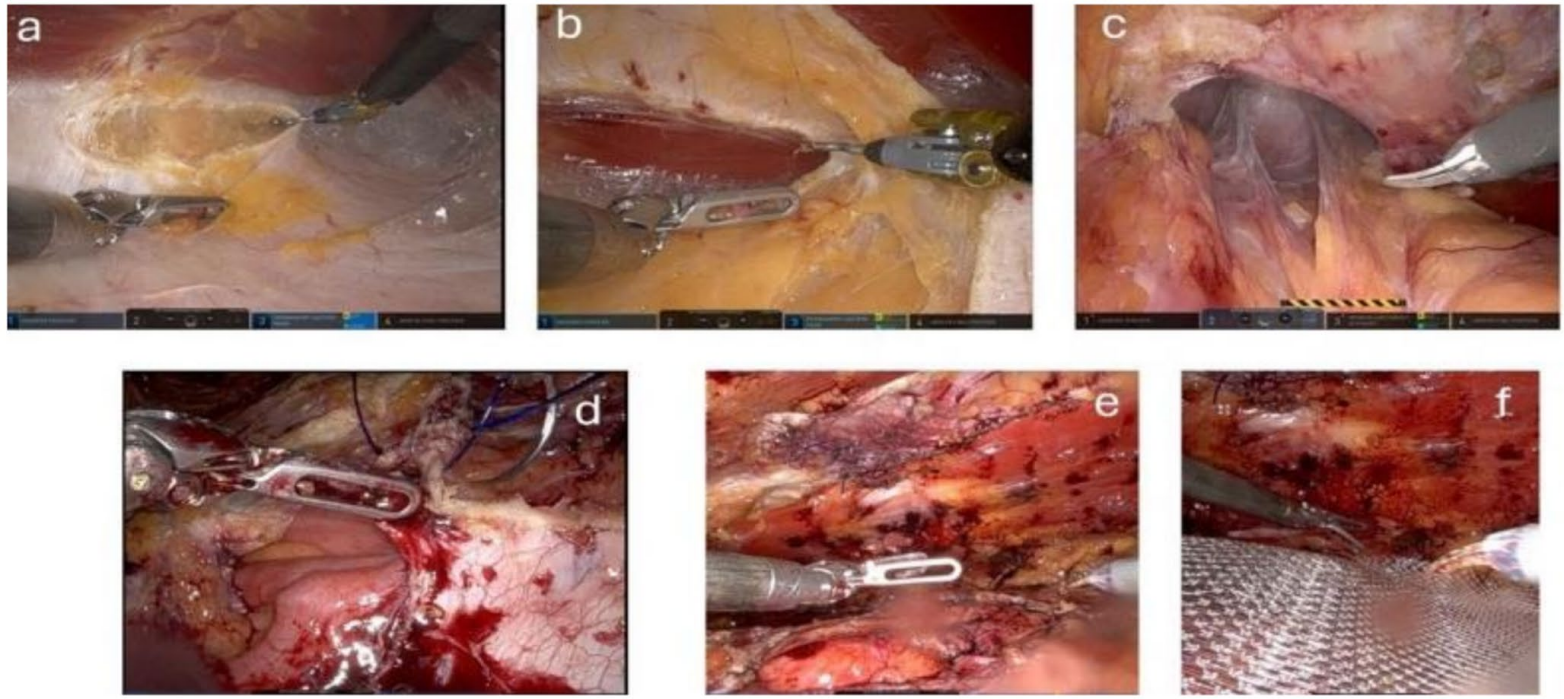
Robotic eTEP (**a**) Transection of the posterior fascial layer on the left side; (**b**) Preperitoneal dissection behind the linea alba and transection of the contralateral posterior fascial layer, opening of the right sided retro muscular space; (**c**) Repositioning of the hernia sac; (**d**) Closure of the hernia defect; (**e**) closed defect; (**f**) Placement of the mesh

The first 11-mm optical assist trocar is inserted into the retro muscular space. The trocar is placed along the rib margins, approximately on the left medio clavicular line. The retro muscular space is insufflated with CO2 at 20 mmHg, allowing for further blunt dissection while preserving the lateral neurovascular bundle. Next, three additional 8-mm robotic trocars are inserted into the retro muscular space along the linea semilunaris at 6 cm intervals and 2 cm away from bony structures. The procedure of robotic eTEP follows the main steps as described for laparoscopic eTEP: (1) Completing the dissection of the retro muscular space. (2) Visualisation of the medial edge of the posterior layer of the rectus sheath and exposing the fusion between the anterior and posterior layers. (3) Incision of the posterior layer of the rectus sheath on the ipsilateral side (Fig. 2a). (4) Preperitoneal dissection behind the linea alba, opening the retro muscular space behind the contralateral musculus rectus abdominis (Fig. 2b). (5) Repositioning of the hernia sac (Fig. 2c). (6) Reconstruction of the Linea alba along with the defect and the posterior layer of the rectus sheath (Fig. 2d). (7) Expanding the retro muscular space behind the contralateral musculus rectus abdominis (Fig. 2e). (8) Placement of the mesh (Soft-Mesh) (Fig. 2f). (9) Placement of Drain if necessary.

### Surgical technique - transversus-abdominis-release

In cases of large or complex defects, a TAR can be performed during the eTEP operation [36]. Large hernias are defined as defects wider than 10 cm or fascial defects affecting more than 25% of the abdominal wall. TAR is also an appropriate method for abdominal wall repair in hernias of the lateral abdominal wall, particularly in the lumbar or flank regions.

Initially, the posterior fascia of the internal oblique muscle is incised medially to the neurovascular bundle to expose the transversus abdominis muscle [37]. Subsequently, the fibres of the transversus abdominis muscle that insert into the posterior rectus sheath are transected. The transversalis fascia along with peritoneum is carefully detached laterally and caudally from the muscle. Following this, the actual hernioplasty can proceed.

### Statistical analysis and propensity score analysis

After the data were supplemented and checked for plausibility, they were analysed via the IBM SPSS Statistics 28 software program.

Propensity score matching (PSM) was then carried out with IBM SPSS Statistics 28 to balance the variables and thus avoid possible confounding. The preoperative variables that could impact the outcome include age, sex, BMI, ASA score, hernia category (M1, M2, M3, M4, and M5), hernia type (primary or incisional), rectus diastasis, hernia defect, risk factors and comorbidities. These variables were selected to calculate the propensity score. A total of 90 patients, 45 per group were included. Matching was performed without replacement to ensure that each case was matched only once. The procedure was optimized to maximize execution performance. Additionally, the order of cases was randomized when matches were drawn. A matching tolerance of 1.0 was set to allow matching even with slight differences between the propensity scores. The maximum agreement between the propensity scores was 1.000, with a median value of 0.364.

Ordinal and nominal scaled data were checked for a normal distribution and reported as the mean values with their standard deviations. If the data were nonnormally distributed, they were reported as the median and interquartile range. Other data, such as dichotomous parameters, are given as percentages. The Kolmogorov‒Smirnov test was used to check for a normal distribution. The statistical methodology used in the data analysis included Student’s t test for continuous variables and the chi-square test for categorical and ordinal variables. If there was no normal distribution, the Mann‒Whitney U test was used. The results with *p* values < 0.05 were considered significant.

## Results

### Population

**Fig. 3:**
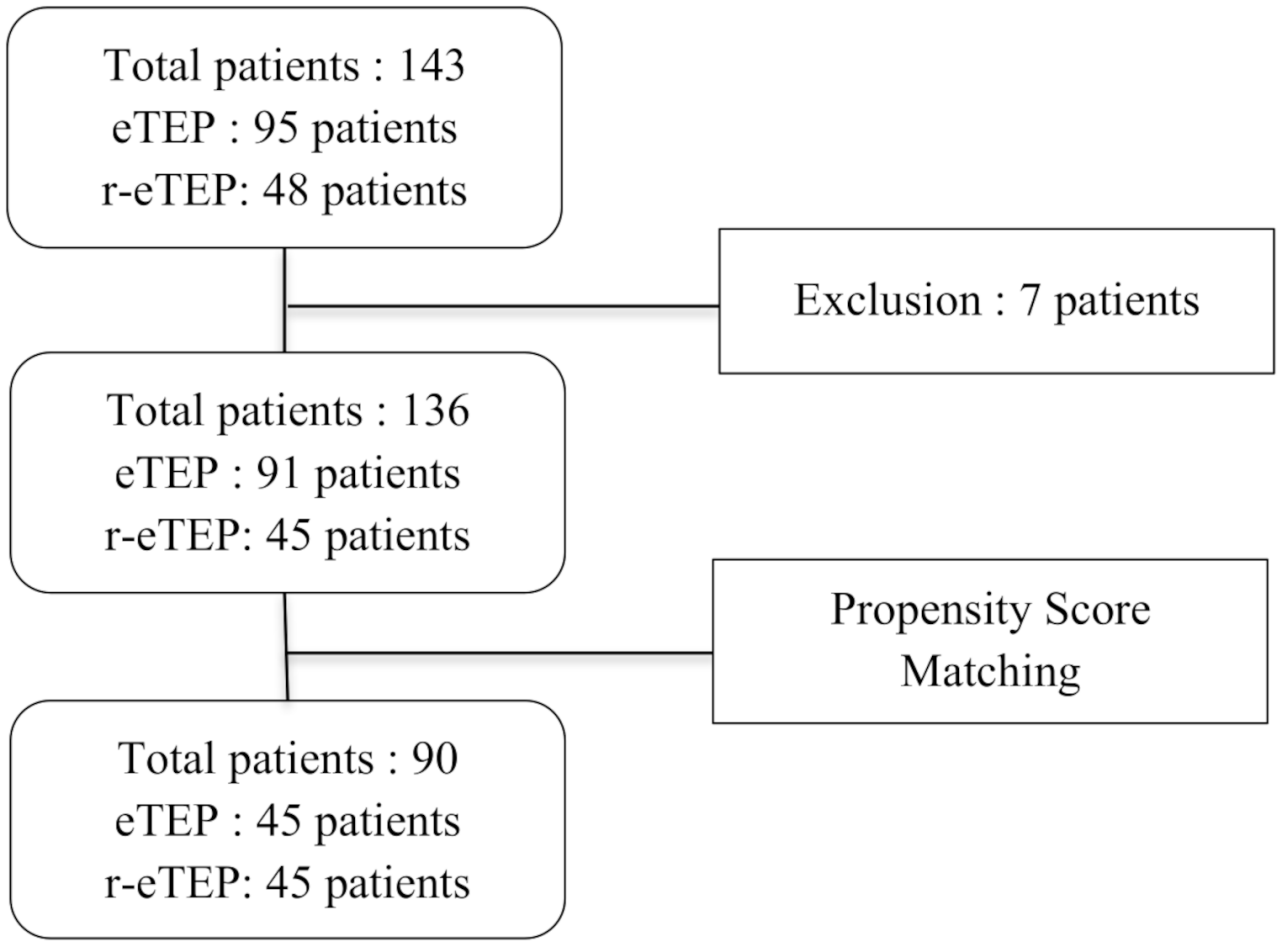
This flowchart illustrates the total number of patients and their distribution between the eTEP and r-eTEP groups. It shows the initial patient count, the number of patients after exclusions, and the final numbers after Propensity Score Matching (PSM)

A total of 143 patients underwent surgery between January 2019 and March 2023, with 95 who underwent eTEP and 48 who underwent r-eTEP. Following the exclusion of seven patients with L2 or L3 hernias according to the exclusion criteria and the application of propensity score matching, a final sample of 90 patients was selected: 45 in the eTEP group and 45 in the r-eTEP group, as shown in Fig. 3.

### Patient demographics

The mean age was 51.40 ± 14.10 years in the eTEP group and 55.09 ± 14.75 years in the r-eTEP group. There were 32 men and 13 women in the eTEP group and 25 men and 20 women in the r-eTEP group. The median BMI was 31 kg/m² (range: 26.5–36.5) in the eTEP group and 29 kg/m² (range: 25–33.5) in the r-eTEP group. The median ASA score was 2 (range: 2–3) in both groups.

No significant differences were found between the two groups regarding smoking, anticoagulation therapy, or obesity, as well as for most comorbidities, such as heart disease, liver disease, or diabetes mellitus. However, lung disease was significantly more common in the r-eTEP group (15.6%) compared to the eTEP group (2.2%, *p* = 0.026).

Regarding hernia types, the EHS classification showed significant differences between the groups. M3 hernias were more frequent in the r-eTEP group (93.3%) compared to the eTEP group (71.1%, *p* = 0.006), as were M4 hernias (31.1% vs. 11.1%, *p* = 0.020). No significant differences were observed for other hernia types (M1, M2, M5). For the width parameter (W1–W3), W1 hernias (defect width < 4 cm) were more common in the eTEP group (75.6%) than in the r-eTEP group (51.1%, *p* = 0.016). W2 hernias (defect width 4–10 cm) were observed in equal proportions in both groups (20%, *p* = 1.000). W3 hernias (defect width ≥ 10 cm) were more frequent in the r-eTEP group (28.9%) compared to the eTEP group (4.4%, *p* = 0.002). Incisional hernias were more frequent in the r-eTEP group (53.3% vs. 46.7%, *p* = 0.002). Hernias with rectus diastasis were significantly more common in the r-eTEP group (51.1%) compared to the eTEP group (4.4%, *p* = 0.001).

All relevant patient demographics are summarized in Table 1.

**Table 1:** Total group - demographics

Variables	eTEP (*n* = 45)	r-eTEP (*n* = 45)	*p*-value
Age (years)	51.40 ± 14.10 *	55.09 ± 14.75 *	0.229
Sex			0,126
Male, *n* (%)	32 (71.1)	25 (55.6)	
Female, *n* (%)	13 (28.9)	20 (44.4)	
BMI (kg/cm^2^)	31 (26.5–36.5) †	29 (25.0–33.5) †	0.090
ASA score	2 (2.0–3.0)	2 (2.0–3.0) †	0.612
Risk factors and Comorbidity			
Smoking, *n* (%)	12 (26.7)	6 (13.3)	0.114
Anticoagulation, *n* (%)	7 (15.6)	4 (8.9)	0.334
Adiposity, *n* (%)	25 (55.6)	21 (46.7)	0.399
Cardiovascular diseases, *n* (%)	19 (42.2)	18 (40.0)	0.830
Pulmonary diseases, *n* (%)	1 (2.2)	7 (15.6)	0.026
Liver diseases, *n* (%)	0 (0.0)	1 (2.2)	0.315
Diabetes, *n* (%)	4 (8.9)	4 (8.9)	1.000
Hernia classification according to EHS			
M1, *n* (%)	2 (4.4)	2 (4.4)	1.000
M2, *n* (%)	20 (44.4)	24 (53.3)	0.399
M3, *n* (%)	32 (71.1)	42 (93.3)	**0.006**
M4, *n* (%)	5 (11.1)	14 (31.1)	**0.020**
M5, *n* (%)	3 (6.7)	2 (4.4)	0.645
W1, *n* (%)	34 (75.6)	23 (51.1)	0.016
W2, *n* (%)	9 (20)	9 (20)	1.000
W3, *n* (%)	2 (4.4)	13 (28.9)	0.002
Hernia type			**0.002**
Primary, *n* (%)	35 (77.8)	21 (46.7)	
Incisional, *n* (%)	10 (22.2)	24 (53.3)	
Hernia type			**0.001**
Rectus diastasis, *n* (%)	2 (4.4)	23 (51.1)	
No rectus diastasis, *n* (%)	43 (95.6)	22 (48.9)	

* = Mean + SD; † = Median + IQR; BMI = Body mass index; ASA = American Society of Anesthesiologists

### Intraoperative findings

The hernia defect was significantly smaller in the eTEP group than in the r-eTEP group (6 [range: 3.6–20] cm² vs. 16 [range: 6.0–74.5] cm², *p* = 0.003). Similarly, the mesh size was significantly larger in the r-eTEP group than in the eTEP group (375 [range: 242.5–450] cm² vs. 420 [range: 355–487] cm², *p* = 0.015). The operation time did not differ significantly between the groups, with a median of 131 min in the eTEP group and 117 min in the r-eTEP group. Transversus abdominis release (TAR) procedures were performed exclusively in the r-eTEP group, with 8 cases (17.8%) compared to none in the eTEP group (0%, *p* = 0.003).

Drainage was placed significantly more often in the r-eTEP group (48.9%) compared to the eTEP group (6.7%, *p* < 0.001). Erythrocyte concentrates (ECs) were required intraoperatively by one patient in the eTEP group, whereas no patients in the r-eTEP group required ECs. There were 2 intraoperative complications in the eTEP group, both involving opening of the peritoneum, while no intraoperative complications occurred in the r-eTEP group.

The intraoperative findings are summarized in Table 2.

**Table 2:** Intraoperative findings

Variables	eTEP (*n* = 45)	r-eTEP (*n* = 45)	*p*-value
Hernia defect (cm²)	6 (3.6–20) †	16 (6.0–74.5) †	**0.003**
Mesh size (cm²)	375 (242.5–450.0) †	420 (355–487) †	**0.015**
TAR, *n* (%)	0 (0.0)	8 (17.8)	**0.003**
Operation time (minutes)	131 (102.5–162.5) †	117 (91.5–147.5) †	0.211
Conversion rate, *n* (%)	0 (0.0)	0 (0.0)	/
Intraoperative complication, *n* (%)	2 (4.4)	0 (0.0)	0.153
Drain placement, *n* (%)	3 (6.7)	22 (48.9)	**< 0.001**
EC, *n* (%)	1 (2.2)	0 (0.0)	0.315

* = Mean + SD; † = Median + IQR; EC: erythrocyte concentrates

### Postoperative outcomes

A significant difference was observed in the postoperative length of stay, with eTEP patients staying longer than r-eTEP patients (3 [range: 2–3.5] days vs. 2 [range: 2–2] days, *p* < 0.001). Postoperative pain on the second day, measured by the VAS score, was similar in both groups.

The postoperative complication rate within 30 days was identical in both groups at 13.3%. No differences were found regarding SSO and SSI rates. In the eTEP group, 4 patients experienced seromas, and 1 patient had a surgical site infection (SSI). In the r-eTEP group, 5 patients developed seromas, and none experienced an SSI. Other complications included persistent pain in one eTEP patient and postoperative ileus in one r-eTEP patient.

There was no significant difference in the incidence of complications between the two groups based on the Dindo classification. One postoperative bleeding at the trocar site in the left lower abdomen, requiring transfusion of two units of packed cells as Category 3 complications occurred in the eTEP group, while category 2 complications were more common in the r-eTEP group.

The postoperative outcomes for all patients are summarized in Table 3.

**Table 3:** Total group– postoperative outcome

Variables	eTEP (*n* = 45)	r-eTEP (*n* = 45)	*p*-value
Admission period (days)	3 (2.0–3.5) †	2 (2.0–2.0) †	**< 0.001**
VAS day 2	1 (0.0–2.0) †	2 (0.0–2.0) †	0.126
Postoperative Complications, *n* (%)	6 (13.3)	6 (13.3)	1.000
SSO, *n* (%)	4 (8.9)	5 (11.1)	0.725
SSI, *n* (%)	1 (2.2)	0 (0.0)	0.315
Others, *n* (%)	1 (2.2)	1 (2.2)	1.000
Clavien Dindo-Classification			0.572
0, *n* (%)	39 (86.7)	39 (86.7)	
1, *n* (%)	2 (4.4)	2 (4.4)	
2, *n* (%)	1 (2.2)	3 (6.7)	
3, *n* (%)	3 (6.7)	1 (2.2)	

* = Mean + SD; † = Median + IQR; VAS = Visual Analogue Scale; SSO = Surgical site occurrence; SSI = Surgical site infection

## Discussion

Minimally invasive techniques such as eTEP and r-eTEP have demonstrated better intraoperative and postoperative results compared to open approaches [9, 10]. r-eTEP repair is a relatively new surgical approach and has shown potential advantages over eTEP in studies conducted thus far [23–26]. However, concerns about longer operative times and higher healthcare costs remain [28]. The number of studies on this topic is limited, and additional studies are therefore needed.

This study aimed to compare the outcomes of eTEP and r-eTEP techniques while addressing gaps in the existing literature, thus providing further evidence for potential treatment strategies.

Preoperative patient characteristics, especially BMI and ASA scores, did not show significant differences between the two groups, unlike other studies that observed significant differences or described higher BMI and ASA scores [31, 32]. However, in our study, the patient data were matched via propensity score matching (PSM) to minimize differences and enable more precise analyses, thus minimizing bias.

A comprehensive analysis of hernia characteristics demonstrated that M3 and M4 hernias according to the EHS classification were significantly more frequently operated on using the r-eTEP technique (M3 *p*-value: 0.006; M4 *p*-value: 0.020). Incisional hernias and hernias with rectus diastasis were also significantly more common in the r-eTEP group. This finding suggest that the r-eTEP technique may be preferable for more managing complex hernias. This aligns with previous studies that noted r-eTEP was used for more challenging cases [32]. This highlights the advantages of the robotic platform, including its precision and ergonomic benefits [38].

In the eTEP group, the median defect size was 6 cm² and the median mesh size required was 375 cm², compared to the r-eTEP group, which had a median defect size of 16 cm² and a median mesh size of 420 cm² (*p*-value = 0.015). These significant differences further support the hypothesis that r-eTEP is better suited for larger and more complex hernia defects, requiring more extensive closures. This is consistent with findings by Lu et al., who also reported a preference for robotic procedures in such cases [32].

The median operation time in this study was 131 min for eTEP and 117 min in r-eTEP, with no statistically significant difference. However, these results contrast with prior studies, which reported longer operative times for robotic techniques [28, 32]. Compared with our results, longer operation times have been reported for the TEP technique, with durations of 168 and 218.9 min, as well as for the r-TEP technique, with a duration of 162.2 min [16, 18, 31]. The shorter operation times observed in this study may reflect the expertise of a single surgeon performing all procedures. The shorter operative times observed in this study may reflect the expertise of a single experienced surgeon performing all procedures. While this ensured consistency and a fair comparison between the two techniques, it also limits the generalizability of the findings, as these results may not be easily replicated by surgeons with varying levels of experience Our results also highlight the benefits of robotics, indicating shorter operative times in r-eTEP than in laparoscopic eTEP, although not significantly. Further studies are needed to validate these trends and evaluate how robotic systems can reduce operative times as their usage becomes more routine [39].

The overall intraoperative complication rate was low in both groups with no significant difference (4.4% for eTEP versus 0% for r-eTEP). However, these complications were not severe and did not require conversion to an open procedure. These findings align with other studies that reported similar results. Belansky et al. reported a relatively low intraoperative complication rate of 2.5% since the introduction of eTEP for ventral hernia repair [16]. Similarly, a meta-analysis reported an intraoperative complication rate of 2% [19]. According to Belyansky et al., no intraoperative complications were described in the r-eTEP group [31]. The low complication rate in both groups confirms the safety and feasibility of the eTEP and r-eTEP techniques, with r-eTEP possibly having an additional safety advantage but without a statistically significant result.

Drains were used more frequently in the r-eTEP group than in the eTEP group. The increased use of the r-eTEP method for complexity of hernias treated with r-eTEP. The decision to place a drain was primarily influenced by intraoperative findings, such as a tendency to bleed, or by the presence of specific risk factors, including therapeutic anticoagulation or diabetes. The size of the hernia defect, however, did not play a role in the decision making process. The higher frequency of drain placement in the r-eTEP may have contributed to the slightly higher incidence of postoperative pain observed with r-eTEP, although the difference was not statistically significant.

The median postoperative hospital stay for robotic eTEP was 2 days (range:2–2), which was significantly shorter than that for laparoscopic eTEP at 3 (range:2–3.5) days (*p* value < 0.001). These results indicate a faster recovery and earlier discharge of patients with the r-eTEP technique Possible explanations for this observation include the enhanced precision and ergonomics of the robotic platform, which may reduce tissue trauma and postoperative discomfort, thereby promoting a faster recovery. This result was observed in the meta-analysis by Peñafiel et al., where the hospital stay was significantly shorter for robotic-assisted compared with laparoscopic repair, with a mean difference of − 1.05 days [40]. In the study conducted by Lu, no relevant difference was observed, with the overall length of stay being low for both techniques (0.2 days for eTEP vs. 0.1 days for r-eTEP) [32]. In other studies, the mean length of stay for r-eTEP was 0.7 days, which was lower than that reported in our study [31, 41]. This difference could reflect variations in practice, with some studies possibly performing these operations on an outpatient basis, while in our department in Germany, we routinely perform inpatient monitoring post-procedure. For instance, in the study by Bauer et al. in Germany, patients stayed in the hospital for an average of 3 days following r-eTEP, which aligns closely with our findings [20]. This suggests that the postoperative length of stay can vary based on country-specific protocols and clinical practices. Further research is required to explore these factors more comprehensively and gain a deeper understanding of the mechanisms underlying this variation.

Our results showed a similar postoperative complication rate within the first 30 days in the eTEP and in the r-eTEP group (13.3%). Similarly, surgical site-specific complications rates, such as SSO and SSI rates, were comparable between the two groups. The only instance of blood transfusion in the laparoscopic group was due to postoperative arterial bleeding at the trocar site in the left lower abdomen. This complication was swiftly addressed at the bedside with suturing. The patient had no known risk factors that could have contributed to the bleeding. In other studies comparing both techniques Lu et al. reported a complication rate of 9.2% for eTEP and 2.3% for r-eTEP [32]. Other studies on r-eTEP also reported a complication rate between 4.9% and 5.4% [20, 31]. Compared with these studies, the postoperative complication rates were comparable. However, this could be influenced by the exclusion procedure and propensity score matching, which allow a better comparison between the two groups and thus increase the validity of our results. In our dataset, 51 patients were excluded from the eTEP group and seven patients from the r-eTEP group had no postoperative complications. Therefore, the observed difference compared to other studies should be interpreted with caution. A direct comparison between the two groups regarding postoperative complications, especially SSO or seroma, is difficult in our case because drains were used significantly more often in the r-eTEP group. This could influence the risk of seroma formation and the validity of the comparison. Further investigations and larger multicenter studies are needed to determine the reasons for these differences.

### Strengths

Our study is characterized by a large patient population, with 96 patients in the eTEP group and 52 in the r-eTEP group. Another advantage of our study is the propensity score matching and the uniform performance of all operations by the same surgeon. This reduces potential confounding factors, which increases the validity and precision of our results and reduces the variability of the results.

### Limitations

This study is limited by its retrospective, non-randomized design and a 30-day observation period, which restricts analysis of long-term recurrence rates. Additionally, reliance on a single experienced surgeon, while ensuring a fair comparison, may limit generalizability, as shorter operative times might not be reproducible by less experienced surgeons. Future studies with longer follow-up and multiple surgeons are needed to confirm these findings.

## Conclusions

The findings of the present study show that the r-eTEP technique may offer potential benefits. In particular, for the repair of more complex hernias such as large hernia defects, incisional hernias, and hernias with rectus diastasis, the r-eTEP technique was more frequently used and has been demonstrated to be the preferred method in this cohort. R-eTEP had a significantly shorter hospital stay compared to e-TEP while intraoperative and postoperative complications were comparable in both groups. However, randomised-controlled studies are necessary to address this potential benefits.

## Data Availability

The data presented in this study are available on request from the corresponding author. The data are not publicly available due to data protection restrictions of our institution and country.
